# Inhibitory Effect of Ginsenoside Rg1 on Vascular Smooth Muscle Cell Proliferation Induced by PDGF-BB Is Involved in Nitric Oxide Formation

**DOI:** 10.1155/2012/314395

**Published:** 2012-03-05

**Authors:** Jing Huang, Li-Sheng Li, Dan-Li Yang, Qi-Hai Gong, Jiang Deng, Xie-Nan Huang

**Affiliations:** ^1^Department of Pharmacology of Zunyi Medical College and the Key Laboratory of Basic Pharmacology of Guizhou, Zunyi 563003, China; ^2^Department of Pharmacology, Mindong Medical School, 65 Manchun Road, Fujian, Fuan, 355017, China

## Abstract

Ginsenoside Rg1 (Rg1) has been reported to suppress the proliferation of vascular smooth muscle cells (VSMCs). This study aimed to observe the role of nitric oxide (NO) in Rg1-antiproliferative effect. VSMCs from the thoracic aorta of SD rats were cultured by tissue explant method, and the effect of Rg1 (20 mg·L^−1^, 60 mg·L^−1^, and 180 mg·L^−1^) on platelet-derived growth factor-BB (PDGF-BB)-induced proliferation was evaluated by MTT assay. The cell cycle was analyzed by flow cytometry. For probing the mechanisms, the content of NO in supernatant and cGMP level in VSMCs was measured by nitric oxide kit and cGMP radio-immunity kit, respectively; the expressions of protooncogene c-fos and endothelial NO synthase (eNOS) mRNA in the VSMCs were detected by real-time RT-PCR; the intracellular free calcium concentration ([Ca2^+^]_i_) was detected with Fura-2/AM-loaded VSMCs. Comparing with that in normal group, Rg1 180 mg·L^−1^ did not change the absorbance of MTT and cell percent of G_0_/G_1_, G_2_/M, and S phase in normal cells (*P* > 0.05). Contrarily, PDGF-BB could increase the absorbance of MTT (*P* < 0.01) and the percent of the S phase cells but decrease the G_0_/G_1_ phase cell percent in the cell cycle, accompanied with an upregulating c-fos mRNA expression (*P* < 0.01), which was reversed by additions of Rg1(20 mg·L^−1^, 60 mg·L^−1^, and 180 mg·L^−1^). Rg1 administration could also significantly increase the NO content in supernatant and the cGMP level in VSMCs, as well as the eNOS mRNA expression in the cells, in comparison of that in the group treated with PDGF-BB alone (*P* < 0.01). Furthermore, Rg1 caused a further increase in the elevated [Ca^2+^]_i_ induced by PDGF-BB. It was concluded that Rg1 could inhibit the VSMC proliferation induced by PDGF-BB through restricting the G_0_/G_1_ phase to S-phase progression in cell cycle. The mechanisms may be related to the upregulation of eNOS mRNA and the increase of the formation of NO and cGMP.

## 1. Introduction

Ginsenoside Rg1 (Rg1) is believed to be one of the main active principles in ginseng (Panax ginseng C. A. Meyer), a traditional Chinese medicine used to enhance stamina and capacity to cope with fatigue as well as physical stress. It has been reported that Rg1 has many beneficial effects on several systems. For example, in cardiovascular system, Rg1 can ameliorate the ventricular remodeling induced by myocardial infarction [1] and the left ventricular hypertrophy induced by abdominal aorta coarctation [2] in rats and protect rat cardiomyocyte from hypoxia/reoxygenation oxidative injury [3]. Also, Rg1 has been reported to inhibit proliferation of vascular smooth muscle cells (VSMCs) induced by tumor necrosis factor-*α* and block the cell cycle in the G1-phase via depressing the signaling pathways of ERK, PI3K/PKB, and PKC [4, 5]. However, cell proliferation is modulated by many factors, more studies should be done to elucidate the action mechanisms of the antiproliferative effect of Rg1.

Nitric oxide (NO) has been known to exert many different vasoprotective effects, such as inhibitions of platelet aggregation [6], leukocyte chemotaxis [7], and endothelial cell apoptosis [8]. For vasculature, mounting evidence has indicated that NO can inhibit VSMC proliferation in vitro and prevent the development of intimal hyperplasia after vascular injury in vivo [9–11]. The inhibitory effect of S-nitroso-N-acetylpenicillamine and sodium nitroprusside, the NO donors, on VSMC proliferation was thought via cGMP-dependent [12] and cGMP-independent [13–15] mechanisms. On the other hand, Rg1 has been reported to cause endothelial-dependent relaxation in the rat aorta [16], enhance endogenous NO production in human umbilical vein endothelial cells [17], rat kidney [18], and in porcine coronary arteries [19]. Interestingly, some pharmacological effects of Rg1 have been attributed to endogenous NO production, such as its improving effect on male mice copulatory behavior [20] and its protection against left ventricular hypertrophy induced by abdominal aorta coarctation in rats [21]. However, whether the antiproliferative effect of Rg1 on VSMC involves in endogenous NO production is unknown.

This study aimed to investigate the possible influence of Rg1 on the cell-cycle progression induced by platelet-derived growth factor-BB (PDGF-BB) and observe the role of NO in Rg1 antiproliferative effect on VSMC.

## 2. Materials and Methods

### 2.1. Materials and Animals

Rg1 (purity >97%) was obtained from Beijing Naturally Occurring Drugs Research Institute, China. Other reagents and their sources were mainly as follows: platelet-derived growth factor-BB (PDGF-BB) (Sigma-Aldrich Co. USA); 3-(4,5-dimethylthiazol-2-yl)-2,5-diphenyltetrazolium bromide (MTT), propidium iodide (PI) dye, and Rnase A (Beijing Solarbio Science Technology Co. Beijing, China); Nitric oxide kit (Jiangsu Beyotime institute of Biotechnology. Jiangsu, China); cGMP radio-immunity kit (Beijing Puerweiye Biological Engineering Co. Beijing, China); *β*-actin primer endothelial nitric oxide synthase (eNOS) primer, c-fos primer and Cyclin D1 primer (Dalian TakaRa Biological Engineering Co. Dalian, China).

Sprague-Dawley rats (150~200 g) were purchased from the Animal Center of Institute of Surgery Research of the Third Military Medical University (Chongqing, China).

### 2.2. Cell Culture and Experimental Design

Rat VSMCs were isolated from rat thoracic aorta using the explanting technique [22]. Briefly, the aortic pieces (about 1 mm^3^) removed the endothelium, and adventitia were cultured in Dulbecco's modified Eagle's medium (Invitrogen Gibco, USA) containing 20% bovine serum, penicillin 100 U/mL, streptomycin 100 *μ* g/mL at 37°C in a humidified atmosphere of 95% air and 5% CO_2_. After about 10 days, the cells were removed by trypsinization and successively subcultured. Cells from passages 3 to 10 were used for the experiments.

The VSMCs were randomly divided into six groups: Normal group (Normal): no drug was added; N + Rg1 group (N + Rg1): Rg1 180 mg·L^−1^ was added in normal cells; PDGF-BB model group (Model): PDGF-BB 25 *μ*g·L^−1^ was added in normal grown cells; Rg1 low-dose group (Rg1-L), middle-dose group (Rg1-M), and high-dose group (Rg1-H): Rg1 20 mg·L^−1^ , 60 mg·L^−1^ , 180 mg·L^−1^ were added, respectively, to the cells treated with 25 *μ*g·L^−1^ PDGF-BB. The above concentrations of PDGF-BB or Rg1 used in each group were final concentration.

### 2.3. Assessment of VSMC Proliferation by MTT Assay

The rat VSMCs were harvested by trypsinization and plated in a 96-well plate at a density of 1 × 10^5^ cells/mL. PDGF-BB and Rg1 were added to each well as the mention in experimental design (PDGF-BB was added at 30 min after the addition of Rg1). Briefly, MTT assays were carried out as follow: VSMCs were grown in 100 *μ*L medium at 37°C under 5% CO_2_ for 24 h in 96-well plates, followed by a incubation with 10 *μ*L MTT (5 g·L^−1^) for 4 h. Then 100 *μ*L (dimethyl sulfoxide) DMSO were added to each well, and an absorbance at 490 nm was read on a Microplate reader.

### 2.4. Cell-Cycle Analysis Using Flow Cytometry (FCM)

VSMCs were seeded in six-well plastic culture dishes at a density of 5 × 10^5^ cells/mL, PDGF-BB and Rg1 were added as MTT assay after cell synchronization and cultured for 24 h. Thereafter, VSMCs were harvested by trypsinization and collected from the six-well plate, placed in Eppendorf tubes and washed with 4°C PBS twice, then fixed with 2 mL 70% ice-ethanol overnight. After centrifugalization, the VSMCs were washed with PBS twice again. Finally, the DNA contents of VSMCs were determined by PI staining and FCM analysis.

### 2.5. Real-Time RT-PCR Analysis of c-Fos, and eNOS mRNA

VSMCs were seeded in 25 mL culture flask at a density of 1 × 10^5^ cells/flask, total RNA was isolated by using the TRIzol (MRC Co., Cincinnati, USA) method after 24 h of PDGF-BB and Rg1 action. Two-step reverse-transcription polymerase chain reaction was used to detect the expression by iCycle iQ Real-Time PCR Detection System (BIO-RAD Co., California, USA), with SYBR Green PCR Master Mix (ABI Co., Foster, USA). The Primers were designed with Express Software and synthesized by TaKaRa Biological Engineering Company (Dalian, China). The following Primers were used: c-fos (GenBank accession no. RA_011723): forward primer (5′–3′)CCC GGC ATC CTT ATT CAA TTA TCA, reverse primer (5′–3′) GTG TTA TCC CAC AGC ATG TCA ACA G; eNOS (GenBank accession no. NM_021838): forward primer (5′–3′) AGC TGG ATG AAG CCG GTG AC, reverse primer (5′–3′) CCT CGT GGT AGC GTT GCT GA; *β*-actin (GenBank accession no. NM_031144): forward primer (5′–3′) GGC CAA CCG TGA AAA GAT GA, reverse primer (5′–3′) CAG CCT GGA TGG CTA CGT ACA. The reaction conditions were 95°C 10 min 1 cycle; 95°C 15 s, annealing temperature 1 min, 40 cycles. The threshold cycle (Ct) values of target genes were normalized with *β*-actin of the same sample, and expressed as were relative to controls.

### 2.6. Measurements of NO Content in Supernatant and cGMP Level in VSMCs

The measurements of NO content in supernatant and cGMP level in VSMCs were undertaken according to the manufacturer's instructions in the assay kits. VSMC culture cell synchronization and experimental design were in accordance with MTT assay.

### 2.7. Measurement of the Intracellular Free Calcium Concentration ([Ca^2+^]_i_) in VSMCs

The cultured VSMCs were centrifuged at 150 g for 5 min at room temperature.The supernatant was discarded, and the cells were washed twice with Hanks solution (in mmol/L : NaCl 137.0, CaCl_2_ 1.3, KCl 5.4, MgSO_4_ 0.8, NaHPO_4_ 0.38, KH_2_PO_4_ 0.44, NaHCO_3_ 4.2, sucrose 5.6, BSA 0.2%; pH 7.4), then the cells were incubated in Hanks solution containing 3 *μ*mol/L Fura-2/AM for 40 min at 37°C, followed by washing twiced to remove the extracellular dye. Finally, the cell number was adjusted to 1 × 10^−6^ cells/mL for measurement of [Ca^2+^)]_i_.

The fluorescence value from 1 mL cell suspension was measured by a RF-5000 dual-wavelength spectrofluorometer (Shimadzu Company, Japan) with excitation wavelengths at 340/380 nm and emission wavelength at 480 nm. The ratio (*R*) of the fluorescence signals at 340/380 (nm) was calculated automatically, and the maximal *R* value (*R*_max⁡_) and minimal *R* value (*R*_min⁡_) were determined by the addition of 10 *μ*L 10% TritonX-100 (final concentration 0.1%) and 10 *μ* L 500 mmol/L EGTA (final concentration 5 mmol/L), respectively. The [Ca^2+^]_i_ was calculated by the formula described before [22] [Ca^2+^)]_i_ = 224 × [(*R* − *R*_min⁡_)/(*R*_max⁡_ − *R*)] × *FD*/*FS*, where *FD* and *FS* are the fluorescence proportionality coefficients obtained at 380 nm under *R*_min⁡_ and *R*_max⁡_ conditions, respectively. The number 224 is the *K*_D_ value of Fura-2/AM. To observe the possible influence of Rg1 on the elevated [Ca^2+^)]_i_ induced by PDGF-BB, Rg1 was added before the addition of this growth factor, and the fluorescence intensity was detected at 5 min after PDGF-BB addition.

### 2.8. Statistical Analysis

All data are presented as mean ± S.E.M. and were analyzed by the one-way ANOVA followed by Student's *t-*test (two-tails) with the SPSS 13.0 software (SPSS Inc, Chicago, Illinois, USA), and significance was set at *P* < 0.05.

## 3. Results

### 3.1. Effect of Rg1 on MTT Assay in Rat VSMCs

**Figure 1:**
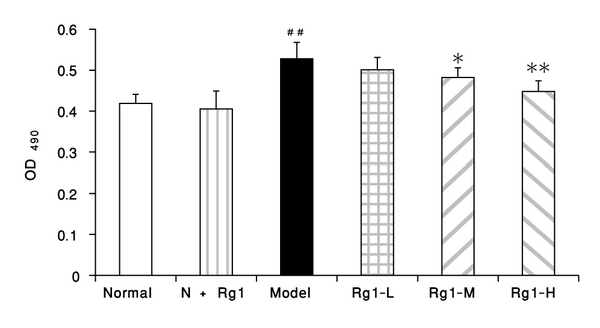
Effect of Rg1 on PDGF-BB-induced VSMC proliferation (MTT assay, $\overset{̅}{x}\pm s$, *n* = 7). The VSMCs were cultured for 24 h. Normal: vehicle; N+Rg1: Rg1 180 mg·L^−1^; model: PDGF-BB 25 *μ*g·L^−1^; Rg1-L: Rg1 20 mg·L^−1^ + PDGF-BB 25 *μ*g·L^−1^; Rg1-M: Rg1 60 mg·L^−1^ +PDGF-BB 25 *μ*g·L^−1^; Rg1-H: Rg1 180 mg·L^−1^ + PDGF-BB 25 *μ*g·L^−1^. Data were mean ± S.E.M. ^##^Significant difference from normal control at *P* < 0.01* significant difference from model control at *P* < 0.05, and ** significant difference from model control at *P* < 0.01.

In normal cells without any treatment of growth factor, Rg1 180 mg·L^−1^ did not change the MTT absorbance, while the addition of PDGF-BB could significantly increase the absorbance in MTT assay (*P* < 0.01), which tended to be inhibited by an addition of Rg1 20 mg·L^−1^ (*P* > 0.05), and was remarkably inhibited by Rg1 60 mg·L^−1^and 180 mg·L^−1^ (*P* < 0.05 and *P* < 0.01), suggesting the antiproliferating effect of Rg1 on VSMCs (Figure 1).

### 3.2. Effect of Rg1 on the Cell Cycle Induced by PDGF-BB in Rat VSMCs

**Figure 2:**
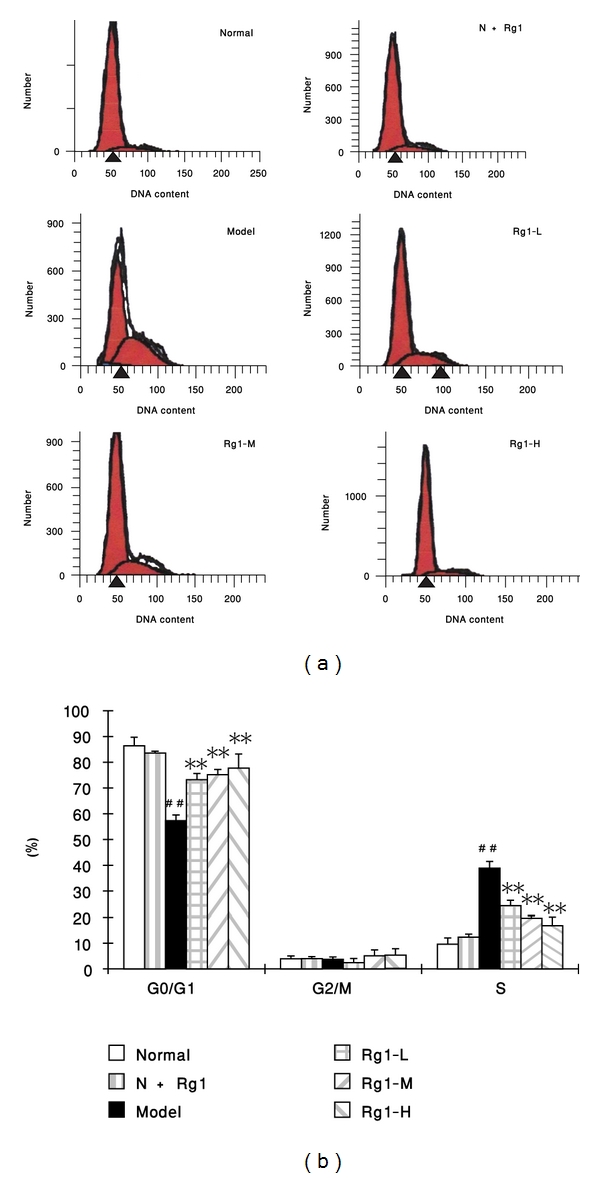
Effect of Rg1 on the VSMC cell cycle in the presence of PDGF-BB ($\overset{̅}{x}\pm s$, *n* = 4 ~ 6,%). A: representative results of flow cytometry measurements to determine the cell-cycle stages of VSMCs; B:percentage of cells in each phase of the cell cycle. Normal: vehicle; N+Rg1: Rg1 180 mg·L^−1^; model : PDGF-BB 25 *μ*g·L^−1^; Rg1-L : Rg1 20 mg·L^−1^ + PDGF-BB 25 *μ*g·L^−1^; Rg1-M : Rg1 60 mg·L^−1^ +PDGF-BB 25 *μ*g·L^−1^; Rg1-H : Rg1 180 mg·L^−1^ +PDGF-BB 25 *μ*g·L^−1^.Data were mean ± S.E.M. ^##^Significant difference from normal control at *P* < 0.01; ** significant difference from model control at *P* < 0.01.

The results shown in Figure 2 demonstrated that Rg1 180 mg·L^−1^ had no any effect on the growth of normal cells, while PDGF-BB could significantly increase the percent of S-phase cells and degrade the *G*_0_/*G*_1_-phase cell percent in the cell cycle (*P* < 0.01) administration of Rg1 (20 mg·L^−1^, 60 mg·L^−1^, 180 mg·L^−1^) at 30 min after PDGF-BB addition could markedly decrease the *S*-phase cell percent and upgrade the *G*_0_/*G*_1_ phase percent in the cell cycle. The data suggested that Rg1 could inhibit the VSMC proliferation induced by PDGF-BB through restricting the *G*_0_/*G*_1_-phase to *S*-phase progression in cell cycle.

### 3.3. Effect of Rg1 on the Expression of c-Fos and eNOS mRNA

**Figure 3:**
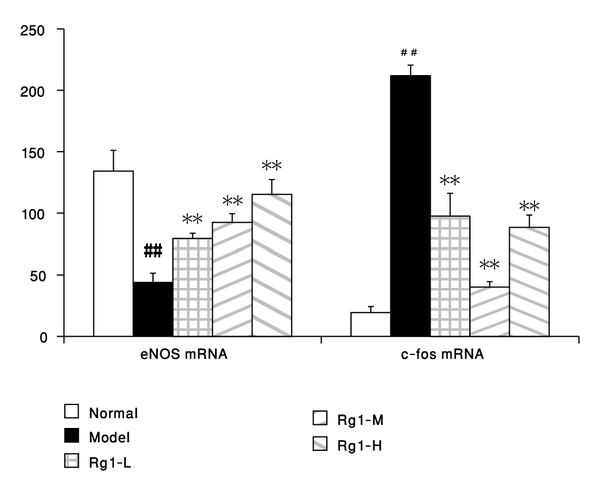
Effects of Rg1 on the changes of expressions of c-fos and eNOS mRNA induced by PDGF-BB in VSMC ($\overset{̅}{x}\pm s,n=3$); real-time RT-PCR analysis of c-fos, and eNOS mRNA were performed after 24 h of PDGF-BB and Rg1 actions in VSMCs. Normal : vehicle; model : PDGF-BB 25 *μ*g·L^−1^; Rg1-L : Rg1 20 mg·L^−1^ + PDGF-BB 25 *μ*g·L^−1^; Rg1-M : Rg1 60 mg·L^−1^ +PDGF-BB 25 *μ*g·L^−1^; Rg1-H : Rg1 180 mg·L^−1^ +PDGF-BB 25 *μ*g·L^−1^. Data were mean ± S.E.M. ^##^Significant difference from normal control at *P* < 0.01; ** significant difference from model control at *P* < 0.01.

The results from real-time RT-PCR assay indicated that PDGF-BB could elevate the c-fos mRNA expression by about 10 times of that in the normal control, and Rg1 could significantly blunt the c-fos expression (*P* < 0.01). On the other hand, addition of PDGF-BB decreased the expression of eNOS mRNA by about 67%, and Rg1 could reverse the decreasing eNOS expression in a concentration-dependent manner (*P* < 0.01) (Figure 3).

### 3.4. Changes of NO Content in Supernatant and cGMP Level in VSMCs

**Figure 4:**
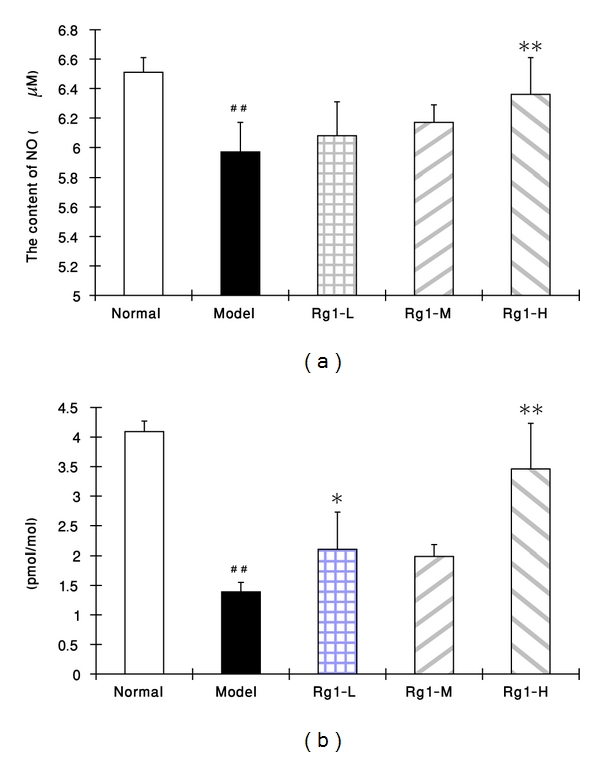
Effects of Rg1 on the NO content in supernatant (a) and cGMP level in cultured VSMCs (b) in the presence of PDGF-BB. VSMCs were cultured for 24 h, the NO content in supernatant and cGMP level in the cells were determined with the corresponding Kits. Normal: vehicle; model: PDGF-BB 25 *μ*g·L^−1^; Rg1-L : Rg1 20 mg·L^−1^ + PDGF-BB 25 *μ*g·L^−1^; Rg1-M : Rg1 60 mg·L^−1^ +PDGF-BB 25 *μ*g·L^−1^; Rg1-H : Rg1 180 mg·L^−1^ +PDGF-BB 25 *μ*g·L^−1^. Data were mean ± S.E.M. ^##^Significant difference from normal control at *P* < 0.01; ** significant difference from model control at *P* < 0.01.

As shown in Figure 4(a), PDGF-BB could significantly degrade the content of NO in the supernatant of the cultured VSMCs (*P* < 0.01), the addition of the low concentration of Rg1 (20 mg·L^−1^) tended to increase the NO content (*P* > 0.05), and the higher concentrations of Rg1 (60 mg·L^−1^, 180 mg·L^−1^) could significantly elevate the content of NO (*P* < 0.05 or *P* < 0.01). Consistent with the changes of NO content in supernatant, the cGMP level in VSMCs was also decreased by PDGF-BB (*P* < 0.01) and was markedly increased by additions of all three concentrations of Rg1 (*P* < 0.05 or *P* < 0.01) (Figure 4(b)).

### 3.5. Change of [Ca^2+^]_i_ in VSMCs by Rg1 treatment

**Figure 5:**
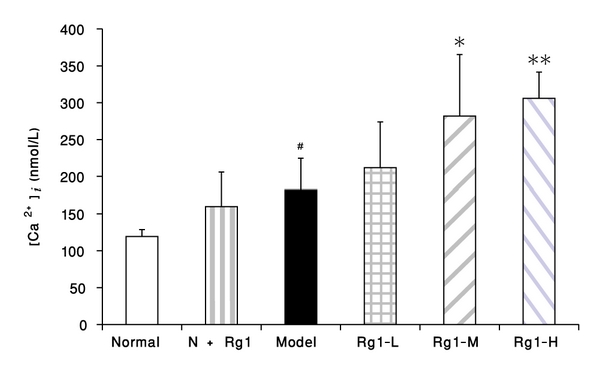
Effect of Rg1 on the intracellular free Ca^2+^ concentration ([Ca^2+^]_i_) of VSMCs. The [Ca^2+^]_i_ was detected after 3 minutes of PDGF-BB addition with Fura-2/AM loaded VSMCs, and Rg1 administrations were performed before 30 minutes of the addition of PDGF-BB. Normal : vehicle; model : PDGF-BB 25 *μ*g·L^−1^; Rg1-L : Rg1 20 mg·L^−1^ + PDGF-BB 25 *μ*g·L^−1^; Rg1-M : Rg1 60 mg·L^−1^ +PDGF-BB 25 *μ*g·L^−1^; Rg1-H : Rg1 180 mg·L^−1^ + PDGF-BB 25 *μ*g·L^−1^. Data were mean ± S.E.M. ^#^Significant difference from normal control at *P* < 0.05; *significant difference from model control at *P* < 0.05, **Significant difference from model control at *P* < 0.01.

The [Ca^2+^]_i_ levels of VSMCs were detected at 3 minutes after the addition of PDGF-BB or at 33 minutes after Rg1-administration. In our experimental conditions, the basal [Ca^2+^]_i_ was about 120 nmol/L in normal VSMCs; it tended to increase by Rg1 180 mg·L^−**1**^ (*P* > 0.05), and was elevated to 182 nmol/L by the addition of PDGF-BB 25 *μ*g·L^−1^(*P* < 0.05). When Rg1 was administered at 30 minutes before PDGF-BB addition, it could cause a further increase in the elevated [Ca^2+^]_i_ induced by this growth factor in a dose-dependent manner (Figure 5).

## 4. Discussion

VSMC proliferation has been known to be an important component of vessel wall remodelling in response to injury, such as after angioplasty and during atherosclerosis formation [23, 24]. The development of highly effective antiproliferative drugs is necessary for the prevention and treatment of hypertrophic vascular diseases. Rg1 has been reported to inhibit TNF-*α*-induced human arterial smooth muscle-cell proliferation and cause cell-cycle arrest in G1 phase [4], and PDGF-BB is known to be a mitogen involved in the development of vascular proliferative lesions observed in atherosclerosis and in restenosis after angioplasty [25, 26]. In the present study, we further certified the antiproliferative effect of Rg1 on the VSMCs from rat-thoracic aorta, using PDGF-BB to induce cell proliferation. Our study also certified that Rg1 could cause *G*_0_/*G*_1_ cell cycle arrest in rat VSMCs, which was very consistent with the report [4]. Furthermore, VSMC proliferation has been known to be promoted by the concerted action of several distinct signal transduction pathways, such as phospholipase C isoforms and mitogen-activated protein kinase (MAPK) cascade [27–29]. The final result of these pathways activation is transcription and activation of the early response genes c-fos and c-jun. Rg1 has been reported to inhibit the PKC and MAPK signalings in TNF-*α*-treated human arterial smooth muscle cells [4, 5]; in this study, we found that Rg1 could also significantly inhibit the elevated c-fos mRNA expression induced by PDGF-BB, which might be the result of the transduction pathway inhibitions.

Because NO has been shown to play diverse roles in the physiology and pathophysiology of the cardiovascular system, including inhibition of VSMC proliferation [9], and Rg1 has been known to promote the endogenous NO production in many tissues [16–21], in the present study, we have emphatically observed the influences of Rg1 on the NO content in supernatant, cGMP level and [Ca^2+^]_i_ in VSMCs, as well as the expression of eNOS in the cells, to clarify the relationship between the antiproliferative effect of Rg1 and NO formation.

It is known that NO stimulates cytosolic soluble guanylyl cyclase to increase cGMP formation, which accompany the activation of cGMP-dependent protein kinase [30, 31]*；*and it has also been known that the NO donors, such as S-nitroso-N-acetylpenicillamine (SNAP) and sodium nitroprusside, inhibit VSMC proliferation by cGMP-dependent [12] and cGMP-independent [13–15] mechanisms. In our study, we found that both NO content in supernatant and cGMP level in VSMCs were degraded by addition of PDGF-BB and elevated by Rg1-treatment. The results seemed to suggest that the increase in cGMP level was from the NO formation after Rg1 addition. Consistent with above results, we also found that Rg1 could significantly upregulate the expression of eNOS in VSMCs in a concentration-dependent manner. Taking the above results together, it appears to be possible that Rg1 promotes the NO formation through upregulating the NOS (at least for eNOS) expression, and to stimulate cytosolic soluble guanylyl cyclase to increase cGMP formation, then the NO itself and cGMP participate the inhibition on VSMC proliferation. It has been reported that in human umbilical vein endothelial cells, Rg1 can downregulate miR-214 (a microRNA related closely to eNOS) expression, leading to an increase in eNOS expression [32]. Whether miR-214 downregulation is involved in the up-regulation of eNOS in this model used in our work remains to be studied.

Notably, the preceding studies indicate that NO inhibits VSMC proliferation by preventing an increase in cell [Ca^2+^]_i_ [33], and Rg1 has been reported to inhibit Ca^2+^ influx in the neurons suffered from hypoxic-ischemic injury [34]. Because vasoconstrictor agonists and growth factors generally cause an increase in cytosolic Ca^2+^ within seconds to minutes [33], we detected the [Ca^2+^]_i_ level of VSMCs at 3 minutes after addition of PDGF-BB. Surprisingly, the result showed that in our experimental conditions, Rg1 (administered at 30 minutes before PDGF-BB addition) could cause a further increase in the elevated [Ca^2+^]_i_ induced by PDGF-BB. Obviously, the increased [Ca^2+^]_i_ could not be attributed to NO formation, and might be the direct action of Rg1 to Ca^2+^ influx or Ca^2+^ release from the endoplasmic reticulum. We conjectured that the NO formation-promoting effect of Rg1 might be not strong enough to reduce the [Ca^2+^]_i_ level of VSMCs at the Ca^2+^-detection time(3 minutes after PDGF-BB addition), and the antiproliferative effect of Rg1 via promoting NO-formation perhaps occurred when larger amount of eNOS expression had been restored. On the other hand, some investigators reported that the Ca^2+^-increasing agents including Ca^2+^ ionophore A23187, ionomycin, cyclopiazonic acid, and di-tert-butylhydroquinone had little effect on proliferation of VSMCs, and they thought that an increase in [Ca^2+^]_i_ *per se* does not appear to be important in VSMC replication [35]. What is the relationship between the Ca^2+^-increasing effect of Rg1 and its VSMC proliferation-inhibiting effect remained to be studied.

It is concluded that one of the mechanisms for Rg1 inhibition on VSMC proliferation is related to the up-regulation of eNOS mRNA and increases the formation of NO and cGMP.

## References

[1] Hin H, Liu Z, Li F (2011). Ginsenoside Rg1 enhances angiogenesis and amelioeates ventricular remodeling in a rat model of myocardial infarction. *Journal of Molecular Medicine*.

[2] Deng J, Lv XT, Wu Q, Huang XN (2009). Ginsenoside Rg1 inhibits rat left ventricular hypertrophy induced by abdominal aorta coarctation: involvement of calcineurin and mitogen-activated protein kinase signalings. *European Journal of Pharmacology*.

[3] Zhu D, Wu L, Li CR (2009). Ginsenoside Rg1 protects rat cardiomyocyte from hypoxia/reoxygenation oxidative injury via antioxidant and intracellular calcium homeostasis. *Journal of Cellular Biochemistry*.

[4] Ma ZC, Gao Y, Wang YG, Tan HL, Xiao CR, Wang SQ (2006). Ginsenoside Rg1 inhibits proliferation of vascular smooth muscle cells stimulated by tumor necrosis factor-*α*. *Acta Pharmacologica Sinica*.

[5] Zhang HS, Wang SQ (2006). Ginsenoside Rg1 inhibits tumor necrosis factor-*α* (TNF-*α*)- induced human arterial smooth muscle cells (HASMCs) proliferation. *Journal of Cellular Biochemistry*.

[6] Radomski MW, Palmer RMJ, Moncada S (1987). Endogenous nitric oxide inhibits human platelet adhesion to vascular endothelium. *The Lancet*.

[7] Kubes P, Suzuki M, Granger DN (1991). Nitric oxide: an endogenous modulator of leukocyte adhesion. *Proceedings of the National Academy of Sciences of the United States of America*.

[8] Tzeng E, Kim YM, Pitt BR, Lizonova A, Kovesdi I, Billiar TR (1997). Adenoviral transfer of the inducible nitric oxide synthase gene blocks endothelial cell apoptosis. *Journal of Surgery*.

[9] Kibbe M, Billiar T, Tzeng E (1999). Inducible nitric oxide synthase and vascular injury. *Cardiovascular Research*.

[10] Tsihlis ND, Oustwani CS, Vavra AK (2011). Nitric oxide inhibits vascular smooth muscle cell proliferation and neointimal hyperplasia by increasing the ubiquitination and degradation of ubch10. *Cell Biochemistry and Biophysics*.

[11] Kapadia MR, Chow LW, Tsihlis ND (2008). Nitric oxide and nanotechnology: a novel approach to inhibit neointimal hyperplasia. *Journal of Vascular Surgery*.

[12] Yu SM, Hung LM, Lin CC (1997). cGMP-elevating agents suppress proliferation of vascular smooth muscle cells by inhibiting the activation of epidermal growth factor signaling pathway. *Circulation*.

[13] Boerth NJ, Dey NB, Cornwell TL, Lincoln TM (1997). Cyclic GMP-dependent protein kinase regulates vascular smooth muscle cell phenotype. *Journal of Vascular Research*.

[14] Cornwell TL, Arnold E, Boerth NJ, Lincoln TM (1994). Inhibition of smooth muscle cell growth by nitric oxide and activation of cAMP-dependent protein kinase by cGMP. *American Journal of Physiology*.

[15] Ishida A, Sasaguri T, Kosaka C, Nojima H, Ogata J (1997). Induction of the cyclin-dependent kinase inhibitor p21(Sdi1/Cip1/Waf1) by nitric oxide-generating vasodilator in vascular smooth muscle cells. *Journal of Biological Chemistry*.

[16] Kang SY, Schini-Kerth VB, Kim ND (1995). Ginsenosides of the protopanaxatriol group cause endothelium-dependent relaxation in the rat aorta. *Life Sciences*.

[17] Leung KW, Cheng YK, Mak NK, Chan KKC, David Fan TP, Wong RNS (2006). Signaling pathway of ginsenoside-Rg1 leading to nitric oxide production in endothelial cells. *FEBS Letters*.

[18] Han SW, Kim H (1996). Ginsenosides stimulate endogenous production of nitric oxide in rat kidney. *International Journal of Biochemistry and Cell Biology*.

[19] Chai H, Zhou W, Lin P, Lumsden A, Yao Q, Chen C (2005). Ginsenosides block HIV protease inhibitor ritonavir-induced vascular dysfunction of porcine coronary arteries. *American Journal of Physiology*.

[20] Wang X, Chu S, Qian T, Chen J, Zhang J (2010). Ginsenoside Rg1 improves male copulatory behavior via nitric oxide/cyclic guanosine monophosphate pathway. *The Journal of Sexual Medicine*.

[21] Deng J, Wang YW, Chen WM, Wu Q, Huang XN (2010). Role of nitric oxide in ginsenoside Rg1-induced protection against left ventricular hypertrophy produced by abdominal aorta coarctation in rats. *Biological and Pharmaceutical Bulletin*.

[22] Li B, Wu Q, Shi JS (2005). Effects of protopine on intracellular calcium and the PKC activity of rat aorta smooth muscle. *Acta Physiologica Sinica*.

[23] Braun-Dullaeus RC, Mann MJ, Dzau VJ (1998). Cell cycle progression: new therapeutic target for vascular proliferative disease. *Circulation*.

[24] Sriram V, Patterson C (2001). Cell cycle in vasculoproliferative diseases potential interventions and routes of delivery. *Circulation*.

[25] Ferns GA, Raines EW, Sprugel KH, Motani AS, Reidy MA, Ross R (1991). Inhibition of neointimal smooth muscle accumulation after angioplasty by an antibody to PDGF. *Science*.

[26] Ross R (1993). The pathogenesis of atherosclerosis: a perspective for the 1990s. *Nature*.

[27] Seger R, Krebs EG (1995). The MAPK signaling cascade. *FASEB Journal*.

[28] Pelech SL, Sanghera JS (1992). Mitogen-activated protein kinases: versatile transducers for cell signaling. *Trends in Biochemical Sciences*.

[29] Pazin MJ, Williams LT (1992). Triggering signaling cascades by receptor tyrosine kinases. *Trends in Biochemical Sciences*.

[30] Moro MA, Russell RJ, Cellek S (1996). cGMP mediates the vascular and platelet actions of nitric oxide: confirmation using an inhibitor of the soluble guanylyl cyclase. *Proceedings of the National Academy of Sciences of the United States of America*.

[31] Sandirasegarane L, Diamond J (1999). The nitric oxide donors, SNAP and DEA/NO, exert a negative inotropic effect in rat cardiomyocytes which is independent of cyclic GMP elevation. *Journal of Molecular and Cellular Cardiology*.

[32] Chan LS, Yue PY, Mak NK, Wong RNS (2009). Role of microRNA-214 in ginsenoside-Rg1-induced angiogenesis. *European Journal of Pharmaceutical Sciences*.

[33] Jeremy JY, Rowe D, Emsley AM, Newby AC (1999). Nitric oxide and the proliferation of vascular smooth muscle cells. *Cardiovascular Research*.

[34] Zhang YF, Fan XJ, Li X (2008). Ginsenoside Rg1 protects neurons from hypoxic-ischemic injury possibly by inhibiting Ca^2+^ influx through NMDA receptors and L-type voltage-dependent Ca^2+^ channels. *European Journal of Pharmacology*.

[35] Shukla N, Jeremy JY, Nicholl P, Krijgsman B, Stansby G, Hamilton G (1997). Short-term exposure to low concentrations of thapsigargin inhibits replication of cultured human vascular smooth muscle cells. *British Journal of Surgery*.

